# Editorial Comment: Luts-V: A new simplified score for assessing lower urinary tract symptoms in men

**DOI:** 10.1590/S1677-5538.IBJU.2020.0278.1

**Published:** 2021-03-25

**Authors:** Ralf Anding

**Affiliations:** 1 University Hospital Basel Dept. of Urology Switzerland Dept. of Urology, University Hospital Basel, Switzerland

## COMMENT

The manuscript features a modified visual scoring system for lower urinary tract symptoms (LUTS) that has a potential for broader use and might be particularly useful for countries with a relevant percentage of individuals with low education levels. According to the World Bank the regional rate (2018) of illiterate people is 6% in Latin America and the Caribbean, 21% in the Middle East and North Africa, 26% in India, 4% in East Asia and the Pacific, and 25% in the Arab World. On average 14% of the World population is illiterate. Therefore, an easy system to characterize urologic symptoms of these patients will facilitate their access to national health systems and appropriate treatment.

According to LUTS and standard instruments Johnson et al. found that patients with limited education or literacy levels demonstrated profound inability to understand the AUA-SS with only 16% able to fully understand the AUA-SS. Moreover, 60% of those who completed the AUA-SS questionnaire reportedly did not understand it (1). In a comment David F. Penson from University of Southern California at Los Angeles replied to the use of these standard instruments: “If we cannot be assured of their validity in patients with low literacy skills, how can we provide these vulnerable patients with optimal care?”

Ten years ago van der Walt et al. developed a new Visual Prostate Symptom Score (VPSS) using pictures rather than words to assess lower urinary tract symptoms (LUTS) and found a significant correlation with the IPSS, Q max. and Qave.. VPSS could be completed without assistance by a greater proportion of men with limited education (2).

This article presents a new visual score (LUTS-V) with some modifications of the VPSS (3). The goal was to further simplify the present pictograms and the authors evaluated the applicability. Despite the respectable intention of this study that finally justified publication, many limitations became apparent during the review process. With respect to the IPSS questionnaire, urinary urgency items were omitted. The pictograms had to be modified to bring the number of day/nighttime micturitions more in line with ICS standards. Finally, the question remains if this modified visual scoring systems adds substantially to the existing literature.

Nevertheless, the improvement of care of this underprivileged and also underreported group of patients is necessary. Further creative studies should add symptoms like urgency to the pictograms that will stimulate the broader use of visual scoring systems. In this regard also cultural aspects must be taken into account. These efforts will hopefully be supported by national health systems.
